# Comparative analyses of DHA-Phosphatidylcholine and recombination of DHA-Triglyceride with Egg-Phosphatidylcholine or Glycerylphosphorylcholine on DHA repletion in n-3 deficient mice

**DOI:** 10.1186/s12944-017-0623-2

**Published:** 2017-12-08

**Authors:** Fang Wu, Dan-dan Wang, Min Wen, Hong-xia Che, Chang-hu Xue, Teruyoshi Yanagita, Tian-tian Zhang, Yu-ming Wang

**Affiliations:** 1 College of Food Science and Engineering, Ocean University of China, No. 5 Yushan Road, Qingdao, 266003 China; 2 Qingdao National Laboratory for Marine Science and Technology, Laboratory of Marine Drugs & Biological Products, Qingdao, Shandong Province 266237 China; 3 Institute of BioPharmaceutical Research, Liaocheng University, Liaocheng, 252059 China; 4 Laboratory of Nutrition Biochemistry, Department of Applied Biochemistry and Food Science, Saga University, Saga, 840-8502 Japan

**Keywords:** DHA-Phosphatidylcholine, Tissue accretion kinetics, Repletion, N-3 Fatty acid deficiency, Weaning

## Abstract

**Background:**

Docosahexaenoic acid (DHA) is important for optimal neurodevelopment and brain function during the childhood when the brain is still under development.

**Methods:**

The effects of DHA-Phosphatidylcholine (DHA-PC) and the recombination of DHA-Triglyceride with egg PC (DHA-TG + PC) or α-Glycerylphosphorylcholine (DHA-TG + α-GPC) were comparatively analyzed on DHA recovery and the DHA accumulation kinetics in tissues including cerebral cortex, erythrocyte, liver, and testis were evaluated in the weaning n-3 deficient mice.

**Results:**

The concentration of DHA in weaning n-3 deficient mice could be recovered rapidly by dietary DHA supplementation, in which DHA-PC exhibited the better efficacy than the recombination of DHA-Triglyceride with egg PC or α-GPC. Interestingly, DHA-TG + α-GPC exhibited the greater effect on DHA accumulation than DHA-TG + PC in cerebral cortex and erythrocyte (*p* < 0.05), which was similar to DHA-PC. Meanwhile, DHA-TG + PC showed a similar effect to DHA-PC on DHA repletion in testis, which was better than that of DHA-TG + α-GPC (*p* < 0.05).

**Conclusion:**

We concluded that different forms of DHA supplements could be applied targetedly based on the DHA recovery in different tissues, although the supplemental effects of the recombination of DHA-Triglyceride with egg PC or α-GPC were not completely equivalent to that of DHA-PC, which could provide some references to develop functional foods to support brain development and function.

## Background

Docosahexaenoic acid (DHA) is highly accumulated in the brain and retina, which is critical for normal nervous development and function [1, 2]. Docosahexaenoic acid can be synthesized from its essential fatty acid precursor, α-linolenic acid (ALA). However, the capacity of brain for synthesizing the long-chain polyunsaturated fatty acids is very limited especially in early life stage, thus DHA is mainly supplied via the uteroplacental circulation during pregnancy and the breast milk during nursing [3]. Many pregnant women cannot intake sufficient n-3 polyunsaturated fatty acids (PUFAs) during pregnancy and lactation in modern western diets, which is likely responsible for the DHA deficiency in developing brains of infants and the increasing incidence of neurological disorders [4, 5]. There have been many nutritional means such as the dietary supplements of ALA and DHA to promote the recovery of organ DHA during the timeframe of gestation and/or lactation [6, 7]. Moreover, particular aspects of neurodevelopment such as synaptic pruning and gliogenesis still continue in childhood, suggesting that the nutrition on brain function is critical during this period [8]. Previous studies showed that direct DHA supplements could increase dendritic spine density and neuritogenesis in the hippocampus of mouse [9]. However, to the best of our knowledge, very few studies have investigated the DHA accumulation kinetics in the brain and other tissues during the childhood.

DHA in natural fish oil is normally esterified to triglyceride (TG) and phospholipid (PL). DHA enriched phosphatidylcholine (DHA-PC) is one of the important forms in DHA-PLs. Several studies have shown that DHA-PC exhibited the higher bioavailability and more effective accumulation in brain compared with DHA-TG [10, 11]. Moreover, dietary ingestion of DHA-PC produced a more significant improvement in cognitive performance and emotional well-being than DHA-TG [9]. This might be attributed to its special molecular form including both DHA and phosphatidylcholine (PC). However, the source of DHA-PC is relatively limited compared to DHA-TG, so we expected that whether recombination of DHA-TG with normal PC (not containing any DHA) could be a substitution of DHA-PC on DHA accumulation in tissues. Interestingly, DHA-PC containing one molar of DHA (sn-2) and one molar of PC could be hydrolyzed into sn-2-lysoPC and free DHA by pancreatic phospholipase A2 in intestine. In addition, triglyceride enriched with high levels of DHA was predominantly hydrolyzed into sn-2-monoacylglycerol and free DHA, and ordinary PC was hydrolyzed to sn-2-lysoPC in the intestinal lumen [12].When re-esterification of absorbed fatty acids in erythrocyte was governed by pure availability of compounds, we expected that the recombination of DHA-TG and ordinary PL (not containing any DHA) could have the same effect as intaking DHA-PC on DHA supplementation. Egg PC is an economical and widely applied functional food supplement, which is rich in saturated fatty acids and monounsaturated fatty acids. α-glycerylphosphorylcholine (α-GPC) is water soluble for its special chemical form with one molecule of choline. The different chemical structure of PC might influence the DHA supplement.

Therefore, the present work was investigated to comparatively analyze the effects of DHA- PC and recombination of DHA-TG with egg PC or α-GPC on DHA repletion by weaning n-3 deficient mice model. The results of this study could provide a meaningful reference to improve n-3 PUFAs deficiency by dietary DHA supplement during childhood.

## Methods

### Preparation of DHA-TG and DHA-PC

DHA-PC was separated following the methods as previously performed [13]. Briefly, total lipids were extracted from squid roe (*S. oualaniensis*) and then mixed with one-fifth volume of 0.15 M NaCl solution. The mixture was placed into a separatory funnel and kept for 24 h to completely clear the bottom (chloroform) phase. The chloroform solution was evaporated to dryness under vacuum. Then phospholipids were separated from neutral lipids and glycolipids by silica-gel column chromatography using sequentially chloroform, acetone, and methanol as eluents. The methanol eluent was collected and DHA-PC was obtained after removal of organic solvent under vacuum. The purity of DHA-PC was confirmed according to the HPLC-ELSD analysis (purity >90%). The fatty acid (FA) composition of the DHA-TG, DHA-PC and egg PC was given in Table 1. Dietary α-GPC (purity >99%), egg PC (purity >95%) and DHA-TG were obtained from Tianjin Bodi Chemical Co., Ltd. (Tianjin, China), Suzhou Fushilai Pharmaceutical Co., Ltd. (Suzhou, China) and Weihai Boow Foods Co., Ltd. (Weihai, China), respectively.

**Table 1:** Fatty acid composition of DHA-TG, DHA-PC and egg-PC

Main fatty acids (%)	DHA-TG	DHA-PC	Egg PC
16:0	0.59	17.51	36.2
18:0	0.34	9.09	12.5
18:1n-9	0.25	8.15	28.9
18:2n-6	0.68	0.28	16.1
18:3n-3	0.42	0.24	0.16
20:1n-9	0.27	9.68	0.18
20:4n-6	0.43	2.78	4.3
20:5n-3	7.82	8.94	nd
22:6n-3	72.56	39.89	0.7
SFAs	12.43	27.77	48.95
MUFAs	5.34	18.82	29.37
N6	1.65	4.28	20.72
N3	80.58	49.13	0.96

nd: trace (< 0.05% of fatty acids)

*SFA* saturated fatty acid, *MUFA* monounsaturated fatty acid, *PUFA* polyunsaturated fatty acid, *N-6* n-6 polyunsaturated fatty acids, *N-3* n-3 polyunsaturated fatty acids

### Animals and diets

**Fig. 1:**
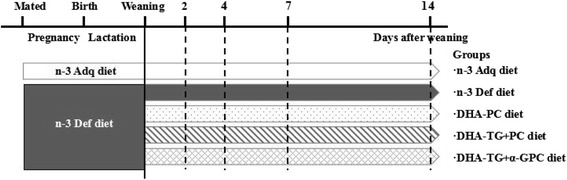
Flow schematic depiction of study design. The dams were sacrificed by decapitation and tissues were collected at days 0, 2, 4, 7, 14 after weaning for fatty acid composition analysis by gas chromatography

All aspects of the animal experiment were carried out in Food Science and Human Health Laboratory of Ocean University of China (Qingdao, P. R. China) and conducted according to guidelines provided by the Ethical Committee of the University (Approval No.: SPXY2015012). The study design was depicted in Fig. 1. Female ICR strain mice aged 7 weeks were purchased from Vital River (Beijing, China) on the second day after conception and immediately randomized into n-3 adequate or n-3 deficient groups, which were fed with n-3 adequate or n-3 deficient diets during pregnancy, respectively. On the first postnatal day, pups were adjusted to 8 per dam and the dams were continually fed with their assigned diets during lactation. All pups were weaned on the 21st postnatal day. The n-3 deficient pups were randomly assigned to four groups as follows: n-3 deficient (n-3 Def) group fed with n-3 deficient diet; DHA-PC group fed with n-3 deficient diet including DHA-PC; DHA-TG + PC group fed with n-3 deficient diet including DHA-TG and egg PC; DHA-TG + α-GPC group fed with n-3 deficient diet including DHA-TG and α-GPC. In addition, the n-3 adequate pups fed with n-3 adequate diet were served as the reference point. The mice were maintained in individual cages under a 12-h light/dark cycle at 23 °C with a 60 ± 10% relative humidity and provided with food and water ad libitum. The basal diets were prepared according to the AIN-93G growth diet and the fatty acid concentration of all diets were summarized in Table 2. The DHA-supplemented diets comprised equal content of DHA + EPA at a dose of 5% of total fatty acid. And the content of PC was adjusted to equimolar ratio of DHA to PC in different DHA-containing diets. The diets were stored at −20 °C and fresh supplies were given to the mice every day. Mice were sacrificed by decapitation after 12-h fast either at weaning or after 2, 4, 7 and 14 days postweaning.

**Table 2:** Ingredient and fatty acid compositions of experimental diets

	Amount (g/100 g diet)				
	n-3 Adq	n-3 Def	DHA-PC	DHA-TG + PC	DHA-TG + α-GPC
Casein, vitamin free	20	20	20	20	20
Sucrose	10	10	10	10	10
Cornstarch	39.749	39.749	39.749	39.749	39.749
Maltose-dextrin	13.2	13.2	13.2	13.2	13.2
Cellulose	5	5	5	5	5
Mineral-salt mix	3.5	3.5	3.5	3.5	3.5
Vitamin mix	1	1	1	1	1
L-Cystine	0.3	0.3	0.3	0.3	0.3
Choline bitartrate	0.25	0.25	0.25	0.25	0.25
TBHQ	0.002	0.002	0.002	0.002	0.002
Fat					
Hydrogenated coconut oil	5.33	5.59	4.22	3.88	4.56
Safflower oil	1.33	1.41	1.41	1.41	1.41
Flaxseed oil	0.34	0	0	0	0
DHA-PC	0	0	1.37	0	0
DHA-TG	0	0	0	0.63	0.63
Egg-PC	0	0	0	1.08	0
α-GPC	0	0	0	0	0.40
Fatty acid composition (%)					
SFAs	73.01	79.79	71.33	70.1	72.7
MUFAs	3.94	3.36	5.61	4.65	4.77
18:2n-6	15.16	14.83	14.51	14.45	14.69
18:3n-3	2.97	0.08	0.1	0.13	0.16
20:4n-6	nd	nd	nd	nd	nd
20:5n-3	nd	nd	1.58	0.43	0.54
22:6n-3	nd	nd	4.18	5.31	5.16
N-6/ N-3	5.10	185.38	2.52	2.52	2.58

nd: trace (< 0.05% of fatty acids)

*TBHQ* tertiary butylhydroquinone, *SFA* saturated fatty acid, *MUFA* monounsaturated fatty acid, *PUFA* polyunsaturated fatty acid, *N-6* n-6 polyunsaturated fatty acids, *N-3* n-3 polyunsaturated fatty acids

Erythrocytes were obtained from trunk blood by centrifugation and washed with phosphate-buffered saline. The cerebral cortex was separated from the whole brain on ice and weighed. The liver and testis from each pup were dissected out and weighed immediately before snap frozen in liquid nitrogen and then stored at −80 °C until further use. Body weights and food intake were recorded every day throughout the experimental period.

### Fatty acid analysis

Total lipids were extracted from samples with a mixture of pentadecanoic acid (15:0) as an internal standard according to the Folch method [14]. The samples with internal standard were extracted by chloroform/methanol (2:1, *v*/v). The lipid extract was evaporated to dryness under nitrogen flux for analysis of fatty acid composition. The total lipids from liver were separated by thin layer chromatography with a mobile phase of petroleum ether: diethyl ether: acetic acid (82:18:1, v/v/v) as previous study [15]. The hepatic phospholipids and triglycerides were scraped off the plate and the obtained lipids were transmethylated to fatty acid methyl esters (FAMEs) with HCl/methanol by shaking at 90 °C for 3 h. The derivatives were extracted by hexane for fatty acids analysis using standard mixture containing 28 kinds of components to identify the retention times. FAMEs were analyzed by an Agilent 6890 gas chromatograph equipped with aflame-ionization detector and an HPINNOW-AX capillary column (30 m × 0.32 mm × 0.25 μm). The detector and injector temperatures were kept at 250 °C and 240 °C, respectively. The oven temperature was increased from 170 °C to 240 °C at 3 °C/min and then held at 240 °C for 15 min. Nitrogen was used as the carrier gas at the flow rate of 1.2 mL/min.

### Statistical analysis

Data were expressed as the mean ± the standard error of the mean (SEM). All the statistical tests were performed with SPSS 18.0 and Figures were made by Graphpad Prism 13.0. Student’s t test was used to compare means between n-3 Def and n-3 Adq groups at 3 weeks of age. Differences between all dietary groups after 3 weeks of age were analyzed by one-way ANOVA. The difference was considered statistically significant when *p* < 0.05.

## Results and discussion

### Time course of fatty acids alteration in cerebral cortex

Previous clinical and preclinical studies suggested that the early postnatal period was a critical interval when insufficient ingestion of n-3 PUFAs might be very detrimental [16]. During this period, particular aspects of neurodevelopment were continuing [17] and the impact of lower n-3 PUFA level was correlated with a greater number of further learning and behavior problems [18]. Therefore, a rapid and efficient recovery of DHA in developing brain was important for optimal function, so we studied the DHA accumulation kinetics in weaning n-3 deficient mice supplemented with different forms of DHA.

**Fig. 2:**
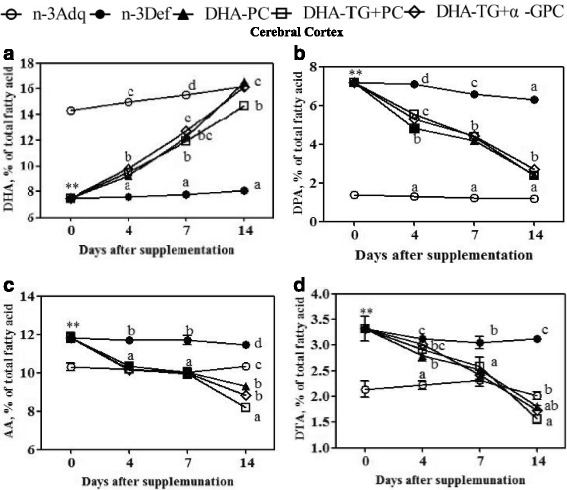
Time course curves of DHA (**a**), DPA (**b**), AA(**c**), DTA(**d**) in cerebral cortex after dietary DHA supplementation from 0 to 14 days. Datas at various time points are given as mean percent ± SEM (*n* = 5 mice per time point). ** *p* < 0.01, significant difference compared to the n-3 Adq group at 3 weeks of age determined by Student’s t-test. Different letters indicate significant difference at *p* < 0.05 among all dietary groups after 3 weeks of age

There were no significant differences observed in food intake, body and tissues weight among all groups during this experiment (data not shown). Interestingly, the initial DHA content in the brain of weaning pups was 14.3% of total fatty acids in n-3 Adq group, but only 7.5% in n-3 Def group, representing a decrease of 47.5% (Fig. 2a; Table 3). As seen in Fig. 2a, the present results also showed a rapid DHA recovery in cerebral cortex, especially the n-3 deficient weaning mice was capable of restoring DHA to the level of the n-3 adequate mice within two weeks by DHA supplementation. All groups including DHA-PC, DHA-TG + PC and DHA-TG + α-GPC exhibited a slight increase of DHA content at 4 post-weaning days but a substantially great amount relative to the n-3 Def group from 4 to 7 days, rising to 12.2, 11.9 and 12.7%, respectively. After 2 weeks, the DHA levels in DHA-PC (16.5%) and DHA-TG + α-GPC (16.1%) groups displayed considerable recovery and nearly reached that of n-3 Adq group (16.2%), which were significantly higher than the DHA-TG + PC group (14.7%). Moreover, the DHA contents of n-3 Def and n-3 Adq groups were only marginally increased within two weeks (n-3 Def group: 7.5–8.1%; n-3 Adq group: 14.3–16.2%). A continually rapid decline in brain DPA was observed in the three DHA-supplemented groups over the subsequent 2 weeks (Fig. 2b; Table 2). The DPA levels of DHA-PC, DHA-TG + PC and DHA-TG + α-GPC groups significantly decreased by 61.3, 62.3 and 57.4% compared to n-3 Def group. The brain DTA and AA patterns for the five dietary groups were quite similar to that of DPA (Fig. 2c and d; Table 3). Compared with n-3 Adq group, the content of DTA and AA in n-3 Def group increased by 55.1 and 14.7% at the weaning day. The AA concentration in the three DHA-containing (DHA-PC, DHA-TG + PC and DHA-TG + α-GPC) mice exhibited a contiguous decline to 9.32, 9.17 and 8.31%, respectively, after DHA supplementation for 4 days. Kitson et al. showed that the DHA content of brain in adult n-3 deficient rat could increase to the normal level after 4 weeks of DHA supplementation [19]. The time courses for the DHA recovery of brain also indicated that the mice required 8 weeks to reach the n-3 adequate level after dietary supplementation of ALA in n-3 deficient mice at 7 weeks of old [20]. Interestingly, the data in present study showed a faster DHA recovery (only 2 weeks required) in the n-3 deficient weaning mice, which was possible that the activity of desaturase enzymes in the young animals was much higher than mature animals [21].

**Table 3:** Brain fatty acid composition

Fatty acid (%)	0 day after weaning		4 days after weaning					7 days after weaning					14 days after weaning				
	n-3 Adq	n-3 Def	n-3 Adq	n-3 Def	DHA-PC	DHA-TG + PC	DHA-TG + α-GPC	n-3 Adq	n-3 Def	DHA-PC	DHA-TG + PC	DHA-TG + α-GPC	n-3 Adq	n-3 Def	DHA-PC	DHA-TG + PC	DHA-TG + α-GPC
14:0	0.55 ± .0.04	0.61 ± 0.04	0.63 ± 0.03	0.65 ± 0.04	0.69 ± 0.03	0.60 ± 0.04	0.61 ± 0.03	0.49 ± 0.01	0.44 ± 0.06	0.45 ± 0.03	0.52 ± 0.03	0.56 ± 0.03	0.70 ± 0.03	0.76 ± 0.02	0.66 ± 0.03	0.66 ± 0.04	0.74 ± 0.05
16:0	22.26 ± 0.27	22.54 ± 0.16	21.23 ± 0.40	21.52 ± 0.30	21.07 ± 0.25	21.48 ± 0.18	21.86 ± 0.17	21.17 ± 0.28	21.09 ± 0.21	20.89 ± 0.17	21.13 ± 0.31	21.54 ± 0.15	20.28 ± 0.31	20.34 ± 0.27	20.41 ± 0.21	20.55 ± 0.23	20.73 ± 0.31
18:0	20.53 ± 0.22	19.95 ± 0.23	19.51 ± 0.31	19.32 ± 0.29	20.06 ± 0.25	19.42 ± 0.25	19.59 ± 0.19	19.38 ± 0.18	19.51 ± 0.21	19.64 ± 0.27	19.84 ± 0.20	19.88 ± 0.23	19.17 ± 0.25	19.35 ± 0.31	19.37 ± 0.22	19.72 ± 0.23	19.61 ± 0.28
SFAs	47.84 ± 0.46	47.42 ± 0.36	47.63 ± 0.36	47.48 ± 0.47	48.14 ± 0.40	47.57 ± 0.54	47.35 ± 0.36	46.55 ± 0.44	46.34 ± 0.33	46.69 ± 0.25	46.13 ± 0.27	45.84 ± 0.39	45.79 ± 0.36	45.92 ± 0.44	45.65 ± 0.25	46.69 ± 0.27	45.69 ± 0.25
16:1n-7	1.32 ± 0.09	1.01 ± 0.12	1.35 ± 0.08	1.21 ± 0.08	1.37 ± 0.09	1.26 ± 0.07	1.37 ± 0.06	1.27 ± 0.03	1.24 ± 0.05	1.44 ± 0.04	1.29 ± 0.10	1.26 ± 0.08	1.38 ± 0.07	1.27 ± 0.05	1.34 ± 0.02	1.34 ± 0.05	1.27 ± 0.01
18:1n-9	14.34 ± 0.24	13.04 ± 0.33	14.96 ± 0.39	14.76 ± 0.27	15.36 ± 0.20	14.75 ± 0.25	14.89 ± 0.23	15.16 ± 0.19	14.98 ± 0.19	15.57 ± 0.28	15.01 ± 0.18	15.08 ± 0.13	15.32 ± 0.32	15.48 ± 0.17	15.96 ± 0.26	15.97 ± 0.18	15.41 ± 0.31
18:1n-7	2.78 ± 0.18	2.89 ± 0.14	2.68 ± 0.15	2.74 ± 0.13	2.69 ± 0.14	2.61 ± 0.11	2.53 ± 0.09	2.55 ± 0.19	2.49 ± 0.16	2.41 ± 0.14	2.82 ± 0.10	2.44 ± 0.14	2.57 ± 0.17	2.35 ± 0.06	2.49 ± 0.11	2.68 ± 0.06	2.53 ± 0.08
MUFAs	22.20 ± 0.47	20.76 ± 0.48	21.86 ± 0.29	21.55 ± 0.33	22.31 ± 0.23	22.51 ± 0.18	22.59 ± 0.22	22.54 ± 0.33	22.86 ± 0.31	22.43 ± 0.27	22.50 ± 0.20	22.58 ± 0.30	22.76 ± 0.30	23.37 ± 0.39	23.07 ± 0.19	23.78 ± 0.27	23.39 ± 0.31
18:2n-6	0.35 ± 0.02	0.46 ± 0.03^*^	0.42 ± 0.03^a^	0.54 ± 0.03^b^	0.61 ± 0.02^c^	0.58 ± 0.01^bc^	0.53 ± 0.02^b^	0.49 ± 0.03	0.52 ± 0.02	0.56 ± 0.03	0.54 ± 0.02	0.51 ± 0.02	0.57 ± 0.02	0.89 ± 0.03	0.42 ± 0.03	0.46 ± 0.02	0.39 ± 0.02
20:4n-6	10.19 ± 0.20	11.97 ± 0.27^**^	10.20 ± 0.16^a^	11.72 ± 0.17^b^	10.34 ± 0.18^a^	10.17 ± 0.16^a^	10.14 ± 0.14^a^	10.04 ± 0.21^a^	11.70 ± 0.25^b^	10.01 ± 0.14^a^	9.96 ± 0.27^a^	9.97 ± 0.11^a^	10.35 ± 0.09^c^	11.45 ± 0.09^d^	9.32 ± 0.10^b^	9.17 ± 0.11^b^	8.81 ± 0.10^a^
22:4n-6	2.13 ± 0.16	3.32 ± 0.25^**^	2.22 ± 0.08^a^	3.12 ± 0.05^c^	2.80 ± 0.11^b^	22.92 ± 0.07^bc^	3.02 ± 0.06^bc^	2.30 ± 0.11^a^	3.05 ± 0.12^b^	2.53 ± 0.24^a^	2.58 ± 0.07^a^	2.42 ± 0.09^a^	2.02 ± 0.08^b^	3.13 ± 0.07^c^	1.79 ± 0.06^ab^	1.86 ± 0.08^ab^	1.73 ± 0.08^a^
22:5n-6	1.40 ± 0.15	7.20 ± 0.19^**^	1.30 ± 0.07^a^	7.01 ± 0.12^d^	4.84 ± 0.09^b^	5.55 ± 0.11^c^	5.30 ± 0.07^c^	1.21 ± 0.12^a^	6.57 ± 0.19^c^	4.19 ± 0.17^b^	4.39 ± 0.18^b^	4.44 ± 0.14^b^	1.19 ± 0.07^a^	6.31 ± 0.12^c^	2.44 ± 0.11^b^	2.38 ± 0.13^b^	2.69 ± 0.09^b^
N-6	15.37 ± 0.25	23.75 ± 0.27^**^	15.04 ± 0.15^a^	23.31 ± 0.19^c^	19.58 ± 0.24^b^	20.01 ± 0.35^b^	19.74 ± 0.17^b^	14.79 ± 0.33^a^	22.89 ± 0.18^d^	18.01 ± 0.31^b^	18.97 ± 0.32^bc^	18.31 ± 0.21^ab^	14.63 ± 0.46^a^	22.77 ± 0.69^b^	14.05 ± 0.39^a^	14.31 ± 0.43^a^	14.17 ± 0.26^a^
22:6n-3	14.30 ± 0.15	7.50 ± 0.16^**^	15.01 ± 0.17^c^	7.60 ± 0.15^a^	9.31 ± 0.19^b^	9.50 ± 0.15^b^	9.82 ± 0.24^b^	15.54 ± 0.25^d^	7.79 ± 0.18^a^	12.21 ± 0.21^bc^	11.90 ± 0.19^b^	12.70 ± 0.20^c^	16.20 ± 0.15^c^	8.10 ± 0.09^a^	16.51 ± 0.09^c^	14.72 ± 0.12^b^	16.12 ± 0.14^c^
N-3	14.50 ± 0.23	8.07 ± 0.19^**^	15.49 ± 0.19^c^	7.75 ± 0.16^a^	9.97 ± 0.18^b^	9.91 ± 0.19^b^	10.32 ± 0.18^b^	16.12 ± 0.23^d^	7.91 ± 0.22^a^	12.87 ± 0.12^bc^	12.40 ± 0.23^b^	13.27 ± 0.15^c^	16.82 ± 0.15^c^	8.26 ± 0.12^a^	17.23 ± 0.13^c^	15.22 ± 0.14^b^	16.75 ± 0.19^c^
PUFAs	29.96 ± 0.63	31.82 ± 0.65	30.51 ± 0.43	31.06 ± 0.51	29.55 ± 0.73	29.92 ± 0.50	30.06 ± 0.39	30.91 ± 0.56	30.80 ± 0.39	30.88 ± 0.43	31.37 ± 0.55	31.58 ± 0.36	31.45 ± 0.47	31.03 ± 0.77	31.28 ± 0.38	29.53 ± 0.45	30.92 ± 0.36
N-6/ N-3	1.06 ± 0.02	2.94 ± 0.04^**^	0.97 ± 0.01^a^	3.02 ± 0.07^c^	1.96 ± 0.04^b^	2.01 ± 0.03^b^	1.91 ± 0.04^b^	0.92 ± 0.01^a^	2.89 ± 0.07^d^	1.40 ± 0.01^b^	1.53 ± 0.01^c^	1.38 ± 0.01^b^	0.87 ± 0.03^b^	2.76 ± 0.07^a^	0.82 ± 0.03^b^	0.94 ± 0.03^b^	0.85 ± 0.02^b^
Total	100.00	100.00	100.00	100.00	100.00	100.00	100.00	100	100	100	100	100	100	100	100	100	100

Only major Fatty acid methyl esters were presented; thus they do not add up to 100%. EPA (20:5n-3) were not detected (<0.01%, trace). Each parameter was presented as the mean ± SEM (*n* = 6). * *p* < 0.05, ** *p* < 0.01, significant difference compared to the n-3 Adq group at 3 weeks of age determined by Student’s *t*-test. Different letters indicate significant difference at *p* < 0.05 among all dietary groups after 3 weeks of age

*SFA* saturated fatty acid, *MUFA* monounsaturated fatty acid, *PUFA* polyunsaturated fatty acid, *N-6* n-6 polyunsaturated fatty acids, *N-3* n-3 polyunsaturated fatty acids

The fatty acid composition analysis of brain showed that there were no significant differences in the total saturated fatty acids, monounsaturated fatty acids and PUFAs among all dietary groups, which was consistent with the previous results [6] (Table 3). The ratios of n-6 / n-3 PUFAs of n-3 Adq group (1.05) was significantly lower than that of n-3 Def group (2.88). The ratios of n-6 / n-3 PUFAs in DHA-containing groups exhibited a considerable ecovery after dietary DHA supplementation and reached the normal level by the end of experiment (Table 3). The present results showed that the DHA content of brain was substituted by n-6 PUFAs were likely due to the competition between the n-3 and n-6 families for elongation and desaturation enzymes [22].

### Time course of PUFAs alteration in hepatic phospholipids and triglycerides

**Fig. 3:**
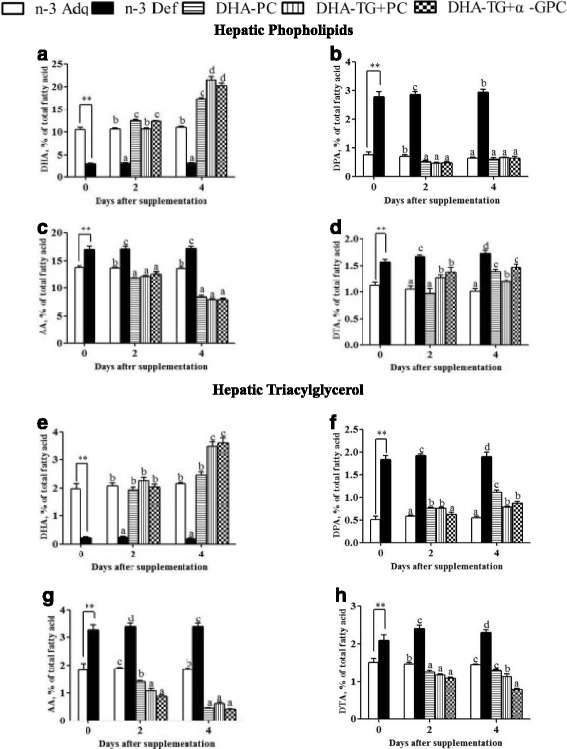
Variation in DHA (**a**), DPA (**b**), AA (**c**), DTA (**d**) in hepatic phosphplipids and DHA (**e**), DPA (**f**), AA (**g**), DTA (**h**) in hepatic thracylglycerols after dietary DHA supplementation from 0 to 4 days. Each parameter is presented as the mean ± SEM (*n* = 6). ** *p* < 0.01, significant difference compared to the n-3 Adq group at 3 weeks of age determined by Student’s t-test. Different letters indicate significant difference at *p* < 0.05 among all dietary groups after 3 weeks of age

The liver was the most rapidly recovery of all tissues examined in this study. The initial DHA values in hepatic PL and TG of n-3 deficient mice were significantly decreased by 73.3 and 89.3%, respectively, compared to n-3 Adq group (Fig. 3a and e). The DHA levels in hepatic TG and PL had fully recovered to the normal values when the n-3 Def mice were supplied with the three DHA-containing diets for two days. There were continually marked increase of DHA levels in hepatic PL and TG after DHA supplementation for 4 days. Interestingly, DHA-TG + PC (21.5%) and DHA-TG + α-GPC (20.24%) groups had higher DHA levels than that of DHA-PC group in hepatic PL (17.3%) (Fig. 3a). For liver TG, the DHA concentration of DHA-PC group (2.46%) was also significantly lower than those of DHA-TG + PC (3.49%) and DHA-TG + α-GPC (3.61%) groups (*P* < 0.05) (Fig. 3e). Previous results found that the ester specific differences were observed only in the livers of normal 10-week-old rats, where the labeled ^4^C-DHA-PL delivered a 2-fold and 1.5-fold higher accretion of radioactivity compared with ^14^C-DHA-TG and ^14^C-DHA-TG + PL, respectively [23]. It was possible that the efficiency of DHA repletion might be influenced by developmental stage of tissue and the initial nutritional status [24]. Compared with the normal adult rats, the mice used in present study were weaning n-3 deficient pups which still in developmental stage and required a mass of n-3 PUFAs to satisfy normal growth. Therefore, we hypothesized that the decreasing DHA accumulation in liver might be beneficial to the availability of DHA for other tissues [11].

Accompanying the loss of DHA, the initial DPA levels in hepatic PL and TG of n-3 Def group were significantly increased by 3.7 and 3.6 folds compared with n-3 Adq group (Fig. 3c and g). After 4 days of DHA supplementation, the DPA content in hepatic PL of DHA-supplemented groups decreased rapidly to nearly 0.5%. And the DPA content in hepetic TG of mice supplied with DHA-PC, DHA-TG + PC and DHA-TG + α-GPC decreased dramatically to 1.11, 0.78 and 0.87%, respectively. In hepatic PL and TG, the AA and DTA levels in n-3 Adq group were significantly lower than those in n-3 Def group.

In hepatic PL, the concentration of AA in DHA-PC, DHA-TG + PC and DHA-TG + α-GPC groups significantly fell from 17 to 8.32%, 7.84%, 7.91%, respectively after 4 days DHA supplementation (Fig. 3c). The AA values in liver TG drastically decreased from 3.26 to 0.45%, 0.61 and 0.42%, respectively after DHA supplementation with DHA-PC, DHA-TG + PC and DHA-TG + α-GPC for 4 days (Fig. 3g). The hepatic DTA pattern for all DHA-supplemented groups was quite similar to that of AA (Fig. 3d and h).

### Time course of PUFAs alteration in erythrocyte

**Fig. 4:**
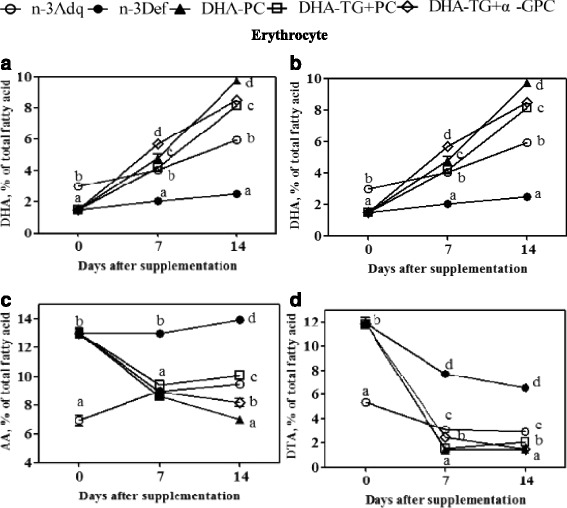
Time course curves of DHA (**a**), DPA (**b**), AA(**c**), DTA(**d**) in erythrocyte after dietary DHA supplementation from 0 to 14 days. Data at various time points are given as mean percent ± SEM (*n* = 5 mice per time point). Different letters indicate significant difference at *p* < 0.05 among all dietary groups after 3 weeks of age

The phospholipids have been observed to be cleaved off into lysophospholipids and free fatty acids by phospholipase A2 mediated partial hydrolysis [25]. Previous reports showed that lyso-DHA-PC combined with albumin was the main source of DHA for the erythrocyte using fatty acid labeled with ^13^C [26]. In erythrocytes, the DHA concentration was significantly lower in n-3 Def group (1.5%) compared to n-3 Adq group (3%), indicating a decrease of 50% at 3 weeks of old (Fig. 4a). Then the DHA value in n-3 Def group rose very slowly to 2.3% over the course of experiment. When the n-3 Def group was supplied with DHA-PC, DHA-TG + PC, DHA-TG + α-GPC during the first week, the DHA content increased rapidly to 4.75, 4.2 and 5.63%, respectively, with a completely recovery compared to n-3 Adq group (4.03%). Then the DHA-TG + PC and DHA-TG + α-GPC groups exhibited a relative increase of DHA level to 8.12 and 8.5% after 2 weeks of supplementation. A faster ascent of DHA from 4.2 to 9.73% was noticed in DHA-PC group. One possible explanation of these results was that a portion of phospholipids might be directly absorbed without phospholipase A2 partial hydrolysis [27]. Ingestion of DHA-PC could increase erythrocyte DHA more effectively compared with DHA-TG [11]. Although the n-3 deficient mice were supplemented with equimolar PC in the three DHA-containing groups, the lyso-DHA-PC concentration in blood of DHA-TG + PC and DHA-TG + α-GPC groups might be less than that in DHA-PC group.

Moreover, DHA in the chemical form of lysophosphatidylcholine was uptake to brain by the primary transporter named MFSD2a across the blood-brain barrier [28, 29], and lyso-DHA-PC represented the major part of DHA supplementation in erythrocyte [30]. A previous study indicated that the DHA level of erythrocyte could been taken as a marker of DHA accumulation in brain during the circulating life span [31]. As seen in this paper, we found a similar time course of DHA reversal in erythrocyte and brain, in which the DHA concentration in DHA-PC group was significantly higher than the other DHA-supplemented groups.

The erythrocyte AA content in n-3 Adq pups (6.93%) was much lower than that of n-3 Def pups (13%, Fig. 4c). The DHA-supplemented groups exhibited a considerable decline of AA after DHA supplementation for 1 week, which nearly reached the level of n-3 Adq group (9%). Then the AA values in DHA-PC and DHA-TG + α-GPC groups decreased gradually to 7 and 8.1% while the DHA-TG + PC group rose slightly to 10% after 2 weeks of repletion. For n-3 Adq group, there was a gradual but continuous increment to 9.48% for AA concentration over the experiment. The initial DPA and DTA levels in n-3 Def groups were significantly higher than that of n-3 Adq group and recovered with a time course similar to that of AA after DHA supplementation (Fig. 4b and d). As previous studies, the n-6 PUFAs content of erythrocyte in n-3 deficient mice would be substitited by DHA after dietary DHA supplementation [32].

### Time course of PUFAs alteration in testis

**Fig. 5:**
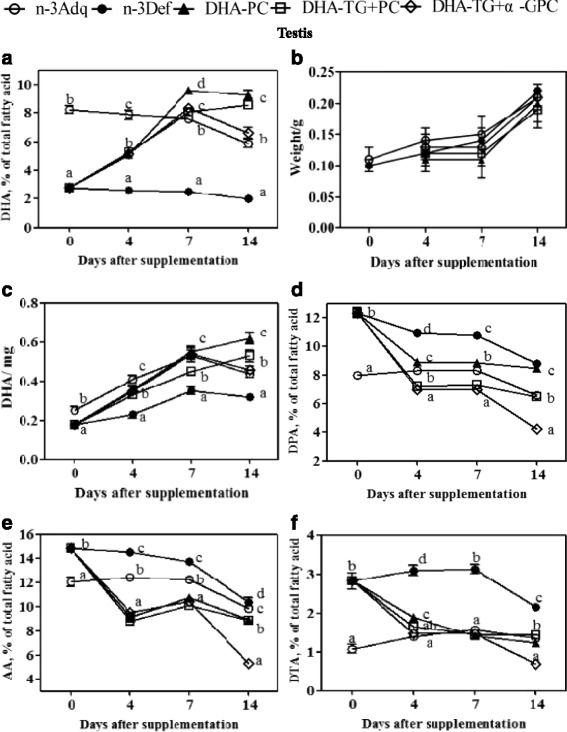
Time course curves of DHA, DPA, AA, DTA (**a**, **d**, **e**, **f**), the weight of testis (**b**) and total quantities of DHA (**c**) in testis after dietary DHA supplementation from 0 to 14 days. Different letters indicate significant difference at *p* < 0.05 among all dietary groups after 3 weeks of age

Testis fatty acid profiles could be influenced by dietary fat and sensitive to n-3 PUFAs [33]. In mice, high levels of DHA, AA, DPA were observed in membrane phospholipids of round spermatids and mature mouse spermatozoa, which suggested an important role for proper spermatogenesis [34]. In this study, the testis DHA concentration in n-3 Def group reduced by 66.2% (*p* < 0.05) compared to n-3 Adq group (8.22%) at the weaning day (Fig. 5a). The DHA levels in dietary DHA-containing groups showed an obvious and continuous increment from 0 to 7 days after weaning. Thereafter, the DHA content in DHA-TG + α-GPC group exhibited a relative decrease from 8.33 to 6.62% from 1 to 2 weeks of DHA supplementation, and DHA-PC group remained the DHA level about 9.25%. Interestingly, DHA-TG + PC group exhibited a slight increase from 8.09 to 8.61% in the DHA value during this period, which nearly reach to that of DHA-PC group. The results might be attributed to the rapid growth of mouse testes from 3 to 4 weeks of age, when the testis weight in DHA-PC, DHA-TG + PC and DHA-TG + α-GPC groups significantly rose by 76.3%, 60.4%, 60.2%, respectively, during this period (Fig. 5b). Further analysis showed that the total amounts DHA in testis of DHA-PC, DHA-TG + α-GPC and DHA-TG + PC groups increased by 93.7, 65.6, and 43.8%, respectively, compared with that of n-3 def group, in which the effect of DHA-TG + PC was similar to that of DHA-PC. Similar to brain and retina, mouse contain excessive amount of PUFAs in testis, with particularly high concentration of DHA, which played an important role for sperm development and function [35]. Previous results showed dietary DHA had the positive effects on male fertility [36]. Dietary supplement with DHA could significantly increase DHA content of testes in delta-6 desaturase-null mice, and as a result that the observed impairment in male reproduction was restored [37]. It was meaningful to explore the time course of testis DHA recovery when the weaning n-3 deficient mice were supplemented with different forms of DHA. The results in this study showed that dietary supplementation with DHA-PC was much more effective for testis DHA accretion than DHA-TG + α-GPC during the developing period.

The initial DPA content of testis in n-3 Adq group (7.98%) was much lower than n-3 Def group (12.33%) (Fig. 5b). The DPA content in DHA-TG + PC and DHA-TG + α-GPC groups displayed a faster decline than that in DHA-PC group within 4 days of DHA supplementation (*p* < 0.05). Thereafter the DPA levels in DHA-PC and DHA-TG + PC groups decreased slightly to 8.46 and 6.49%, respectively, while DHA-TG + α-GPC group exhibited a rapid decline to 4.26% by the end of DHA supplementation. The time course patterns of AA and DTA in testis were similar to that of DPA as seen in Fig. 5c and d.

## Conclusion

The present study investigated the recovery kinetics of tissue DHA after supplementation of DHA-PC and the recombination of DHA-TG with egg PC or α-GPC in weaning n-3 deficient mice induced by maternal dietary deprivation of ALA during pregnancy and lactation. Results showed that dietary DHA supplementation could rapidly recover the DHA concentration of tissues in n-3 deficient mice during the childhood, in which DHA-PC exhibited the optimal efficacy on DHA repletion. Interestingly, the hepatic DHA levels of DHA-TG + PC and DHA-TG + α-GPC groups were significantly higher than that of DHA-PC group after short-term DHA supplementation for 4 days. In addition, DHA-TG + α-GPC exhibited the greater effect on DHA accumulation than DHA-TG + PC in cerebral cortex and erythrocyte, which was similar to DHA-PC. Conversely, DHA-TG + PC was more effective on DHA repletion compared with DHA-TG + α-GPC in testis. Therefore, DHA-PC could not be completely substituted by the recombination of DHA-TG and ordinary PC (not containing any DHA) for DHA supplementation. These findings could pave the way for dietary DHA supplementation in n-3 deficiency conditions especially during childhood, which could provide some references to develop functional foods to support brain development and function.

## Abbreviations

AA: Arachidonic acid (20:4n6)
ALA: a-linolenic acid (18:3n3)
DHA: Docosahexaenoic acid (22:6n3)
DPAn-6: Docosapentaenoic acid (22:5n6)
DTA: Docosatetraenoic acid (22:4n6)
n-3 Adq: n-3 adequate
n-3 Def: n-3 deficient
PC: Phosphatidylcholine
PL: Phospholipid
PUFAs: Polyunsaturated fatty acids
TG: Triglyceride
α-GPC: α-Glycerylphosphorylcholine
